# Regular Meeting, November 9, 1874

**Published:** 1874-11-15

**Authors:** Ralph E. Starkweather


### Regular Meeting, November 9, 1874.

*Reported by Ralph E. Starkweather, M.D.*

THE President, Dr. Bartlett, in the chair. After the usual preliminaries, a motion of suspension of the rules was carried, in order to receive a communication from a Committee of the Chicago Medical Society, in regard to a joint donation of money in temporary support of the widow and young son of a physician who had died soon after the great calamity of 1871. The subject was referred to a committee to report at the next meeting.

Dr. Etheridge, a member of the section on materia medica and therapeutics, presented a very full report, including translations and various interesting items from recent publications. Attention was first called to the new Brazilian sudorific and sialagogue,

### JABORANDI.

This is a South American product; the leaves and small twigs are the parts of the plant used. The leaves have an odor and a bitter taste, but do not appear to have any alkaloids. Sixty to ninety grains of the leaves and twigs may be infused in a cup of water. "When taken, a drenching perspiration, or more properly, sweating, lasting four or five hours, necessitating several changes of clothes, will follow. At the same time an abundant salivary and bronchial secretion supervenes, which may even exceed two pints in quantity."

### AMYL HYDRIDE.

The amyl hydride was exhibited to the Society, and was considered to be innocuous when applied to the skin or mucous membranes. If swallowed it is quickly converted into vapor. It is a remarkable solvent.

A solution containing one scruple of iodine to the ounce of amyl hydride, is very simple, painless, and effective, when applied to bad smelling sores, ulcers, wounds, and to cancer: a very quick disinfectant. Common oils, spermaceti, and other similar oily substances, make ready solutions in amyl hydride.

Burns may be treated with success by using such a solution.

### NITRITE OF AMYL.

After explaining the mode of making the nitrite of amyl, and exhibiting a sample thereof, it was described as diminishing arterial blood tension, as though the vaso-motor nerve supply were paralyzed or cut off. It is a remedy indicated in all cases of cerebral anæmia—in epilepsy when due to that cause. If employed for this purpose it should be given at the commencement of the attack, by inhalation of three or four drops placed in a small vial. In syncope and hemicrania, and chloroform narcosis, amyl has been of benefit. A case reported by Dr. Jenks was cited, of puerperal eclampsia, successfully treated by the nitrite, before and after labor, administering three or four drops by inhalation.

### CARBOLIC ACID HYPODERMICALLY.

Passing next to the subject of carbolic acid hypodermic injections, several cases lately reported were cited, in which results very surprising and satisfactory had been attained. In all forms of parenchymatous inflammation it is an active antiphlogistic—the insertion should be made where the lymphatics bring the acid in direct contact with the inflamed tissue. The strength should be two per cent. of pure acid in water. A solution of one per cent., the injections being repeated four or six times, afforded relief in two cases of erysipelas.

FISSURES IN ANO TREATED BY IODOFORM.

As a local anæsthetic it allays the spasm of the sphincter during defecation; while it favors cicatrization by neutralizing the irritating effects of fæcal matter which may remain on the ulcerated surface. It is used as an ointment, one part to three parts of lard, applied twice daily, on a small piece of charpie. Cure may be expected to follow within twenty days.

BROMIDE OF POTASSIUM.

This salt has been employed by Dr. Bernard, in Algeria, in the treatment of engorgements of the spleen, due to intermittent fever; complete resolution may be secured, the treatment being rarely prolonged beyond thirty days. Hypertrophies of the liver yield just as readily, or at least are very much improved. The dose of the bromide is fifteen grains daily, in one potion, in an infusion of orange leaves, etc., for fifteen to twenty days. In one case, however, during ten days, forty-five grains were given daily, in one potion, followed by cure.

An article by Dr. Chauppe, on "The Experimental Study of the Action of Ipecac," translated by Dr. Etheridge, was then read. It discussed the action of ipecac in arresting diarrhœa.

At a former meeting, an article upon its employment in phthisical sweats had been read. No attempt will be made to give an abstract of this most valuable paper, partly on account of its length, but chiefly because the argument and details would be injured by being at all abbreviated. Dr. Simons reported, verbally, a case of compound comminuted fracture of the right parietal bone, complicated by the protrusion of at least half an ounce of cerebral matter. The patient was a boy of the age of three years. Half an ounce of the brain matter, and a piece of bone one-half by three-quarters of an inch in size, were removed, the depressed bone was elevated, and the lips of the scalp wound were united by sutures. At no time has there been loss of sense, sensation, intellection or motion. The boy is doing well, at the present time, four days after accident.

Dr. Hollister narrated a similar case that had occurred in his practice a number of years ago. The patient was a boy twelve years of age, who had been kicked by a horse. There was a crucial fracture of the parietal bone, near the frontal bone; the meninges were lacerated, and, with the brain substance, protruded from the wound. Six drachms of cerebral matter were removed, and a piece of the parietal bone, one and a half by one and three-quarter inches in size. The wound was closed and compresses applied. Recovery was perfect. The boy never lost consciousness, nor was there failure of the mental powers. He was, however, pretty irritable in temper for a short time.

The motion to reconsider the vote adopting the report and resolutions offered at the last meeting, concerning the relations between physicians and pharmacists, was taken from the table, and, on motion, was laid upon the table indefinitely; thus sustaining the endorsement of them made by the previous meeting.

The Society then adjourned.

